# Co-Prescription Trends in a Large Cohort of Subjects Predict Substantial Drug-Drug Interactions

**DOI:** 10.1371/journal.pone.0118991

**Published:** 2015-03-04

**Authors:** Jeffrey J. Sutherland, Thomas M. Daly, Xiong Liu, Keith Goldstein, Joseph A. Johnston, Timothy P. Ryan

**Affiliations:** 1 Lilly Research Labs IT, Eli Lilly and Company, Indianapolis, Indiana, United States of America; 2 Pathology and Laboratory Medicine Institute, Cleveland Clinic, Cleveland, Ohio, United States of America; 3 Drug Disposition and Toxicology, Eli Lilly and Company, Indianapolis, Indiana, United States of America; 4 Global Patient Outcomes and Real World Evidence, Eli Lilly and Company, Indianapolis, Indiana, United States of America; 5 Sano Informed Prescribing, Brentwood, Tennessee, United States of America; Penn State College of Medicine, UNITED STATES

## Abstract

Pharmaceutical prescribing and drug-drug interaction data underlie recommendations on drug combinations that should be avoided or closely monitored by prescribers. Because the number of patients taking multiple medications is increasing, a comprehensive view of prescribing patterns in patients is important to better assess real world pharmaceutical response and evaluate the potential for multi-drug interactions. We obtained self-reported prescription data from NHANES surveys between 1999 and 2010, and confirm the previously reported finding of increasing drug use in the elderly. We studied co-prescription drug trends by focusing on the 2009-2010 survey, which contains prescription data on 690 drugs used by 10,537 subjects. We found that medication profiles were unique for individuals aged 65 years or more, with ≥98 unique drug regimens encountered per 100 subjects taking 3 or more medications. When drugs were viewed by therapeutic class, it was found that the most commonly prescribed drugs were not the most commonly co-prescribed drugs for any of the 16 drug classes investigated. We cross-referenced these medication lists with drug interaction data from Drugs.com to evaluate the potential for drug interactions. The number of drug alerts rose proportionally with the number of co-prescribed medications, rising from 3.3 alerts for individuals prescribed 5 medications to 11.7 alerts for individuals prescribed 10 medications. We found 22% of elderly subjects taking both a substrate and inhibitor of a given cytochrome P450 enzyme, and 4% taking multiple inhibitors of the same enzyme simultaneously. By examining drug pairs prescribed in 0.1% of the population or more, we found low agreement between co-prescription rate and co-discussion in the literature. These data show that prescribing trends in treatment could drive a large extent of individual variability in drug response, and that current pairwise approaches to assessing drug-drug interactions may be inadequate for predicting real world outcomes.

## Introduction

Drug prescribing in the US is tracked by both public and private institutions using a variety of sources [1, 2]. The ‘most prescribed’ drug lists compiled using these sources typically rank pharmaceutical usage based on overall drug sales and dispensing data, and show that medication use is steadily rising [3]. The effects of drug-drug interactions on drug exposure are usually measured in controlled clinical trials, focusing on interactions predicted from preclinical metabolism studies or projected concomitant drug usage in the intended patient population. These biopharmaceutical clinical trials are typically quite small, testing the drug in development as either a perpetrator or victim of a second drug, with exposure of each drug in blood being the parameter most often measured. Using information from these small studies, it is difficult to determine if theoretical interactions produce actual clinical effects [4]. Furthermore, drug-drug interaction literature and clinical studies focus almost exclusively on pair-wise drug combinations. Given that patients are routinely taking multiple medications, we must better understand drug co-prescribing complexities and their contribution to the heterogeneity of treatment effect if we are to tailor medication treatment to individual patients [5].

A fundamental tenet for pharmaceutical tailoring in medicine is that individual variability in drug response must be considered when treating disease. Variability in response is a product of environmental and genetic factors. The underlying genetic factors are fixed for each patient at birth, whereas environmental components are dependent upon patient and prescriber behaviors relative to disease prevention and management. Unlike genetic factors, environmental factors are potentially controllable, making them viable targets for treatment modification. For example, a key component of disease management is drug selection by prescribers. Drug selection depends upon the potential benefits, risks and cost, which in turn are greatly impacted by drug options, reimbursement, formularies, etc. When multiple physicians are involved in prescribing, treatment regimens can become quite complex, often exposing patients to potentially harmful drug interactions [6], especially when multiple physicians prescribe medications to the same patient [7]. Adverse drug events rise with increasing numbers of prescribing physicians, which is a very important risk factor for potentially inappropriate drug combinations [8]. Avoidable medical costs in the US related to mismanaged polypharmacy in the elderly and prescription errors are estimated at $1.3B and $20B, respectively [9]. An Italian study estimates that 4% of drug-related emergency department visits are caused by drug-drug interactions, and that approximately half of these lead to hospitalization [10].

Polypharmacy appears to be a significant source of medication errors, and a lack of knowledge about complex drug interactions can be a contributor to such errors [11]. Over the years, many systems have been developed to aid prescribers in identifying potentially harmful drug combinations for patients taking multiple medications. These systems manifest in the form of drug alert tools, such as the ‘Drug Interactions’ feature on Drugs.com. However, the drug-drug interaction knowledge that underscores these drug alert systems is largely theoretical and very few alerts have been prospectively validated in clinical pharmacokinetic studies. Retrospective drug-drug interaction studies have shown that more than 60% of drug substrates show significant exposure increases when co-administered with a metabolic inhibitor of that substrate [12]. In a multinational study, 28% of US adults with complex healthcare needs who were taking multiple medications reported that neither a pharmacist nor a physician had reviewed their prescriptions in the past year, demonstrating that awareness is lacking at many levels. In all 11 countries examined in this study, the number of medical errors increased with the number of attending physicians [13].

Recent data mining studies have shown that drug interactions are indeed clinically meaningful and likely more prevalent than those reported using drug interaction sources used herein and by prescribers. Finding clinically meaningful drug interactions in a real world setting requires novel data-mining methodologies [14]. In a comprehensive study, Tatonetti et al. mined the US FDAs Adverse Event Reporting System AERS for side effect profiles. Using an empirical approach, they found a strong synergistic glucose raising effect when pravastatin and paroxetine were co-administered [15]. Conversely, Zhao et al. used this same data source and found that when rosiglitazone was co-administered with exenatide, fewer reports of myocardial infarction were observed [16]. Collectively, these data demonstrate that yet undiscovered drug interactions are likely numerous and that in addition to adverse drug interactions, the addition of a second drug could not only decrease the effectiveness or increase the toxicity of the first drug, but enhance the effectiveness or mitigate the toxicity of the first drug.

Patient responses to drugs are highly variable, even when the same medications are administered to patients with similar disease presentation. Variability in the elderly is even more complex, where age dependent changes in comorbidity, disability, polypharmacy, and composition and function of the body are particularly important [17]. A key step toward understanding patient variability is to understand the various factors that can contribute to heterogeneity in response. Key components include age, genetics, disease state and comorbidities, overall health and health management, and the collective pharmacopeia of co-administered medications. We have used the National Health and Nutrition Examination Survey (NHANES) database as a source of drug prescribing data to investigate medication co-prescribing habits and how they may contribute to multi-drug interactions, which are likely key components of variability in efficacy and safety outcomes. We have evaluated both population and individual components of NHANES data in combination with known drug-drug interaction data to illustrate the complexity in prescribing habits and how these habits may be contributing to patient variability using an elderly patient cohort. The trends in co-prescribing habits identified from this data serve as a first step toward understanding variability in complex drug response.

## Materials and Methods

### Ethics statement

The NHANES studies are approved by the National Center for Health Statistics Research Ethics Review Board (http://www.cdc.gov/nchs/nhanes/irba98.htm; accessed 10 November 2014), and participants provided informed consent before data collection. Because the current study involves secondary analysis of these publicly available, de-identified data, no further IRB approval was sought.

### NHANES data releases

The NHANES database is the product of an ongoing program sponsored by the Center for Disease Control and Prevention that gathers health data on individuals representative of the US population, oversampling for certain ethnicities and age groups. We analyzed publicly-available NHANES data releases for the period 1999 to 2010 from the National Center for Health Statistics, containing self-reported medication usage. These data, in combination with provided sampling weights, were used to produce associations representative of the non-institutionalized civilian US population (http://www.cdc.gov/nchs/nhanes.htm; accessed 30 September 2014). Medication usage was ascertained during home interviews where subjects were asked to list names and dosages of all current medications and to show containers for verification. The 2009–2010 survey, the primary focus of this work (RXQ_RX_F; http://wwwn.cdc.gov/nchs/nhanes/2009-2010/RXQ_RX_F.htm; accessed 30 September 2014), consisted of 10,537 subjects, of which 1,523 were ≥65 years of age. This subset of subjects is referred to as ‘elderly’ throughout this manuscript. This particular NHANES data release included information gathered on 690 specified drugs (i.e. excluding non-specific drugs such as ‘ANTIHISTAMINES—UNSPECIFIED’) organized into 17 medication classes (RXDICN1A), each of which contained multiple sub-classes of medications (RXDICN1B and RXDICN1C). All upper-case variables used here were taken from the NHANES codebook [18]. When analyzing co-prescription of specific drug pairs, we also analyzed data from the 2007–2008 survey (RXQ_RX_E) in order to increase sample size. This survey consisted of 1,556 elderly subjects and contained information on 700 specified drugs.

All analyses adjusted the statistical weight of each subject according to the variable WTINT2YR in all estimates of drug usage, drug pair usage, prevalence of drug-drug interactions, etc. We included all reported drug usage, whether the container was seen or not (i.e RXQSEEN 1 or 2). For 78% of reported drug usage in the 2009–2010 survey, the container was seen by the interviewer.

### Drugs.com alert analysis

Using the 2009–2010 survey, we identified 672 drug pairs with projected co-prescription ≥ 0.1% in the US population (and actually reported by 5 or more NHANES subjects). Each pair was queried for potential drug interactions using the Drugs.com website. Drug-drug interaction severity and clinical relevance criteria were classified into the fields of Major, Moderate, or Minor according to database creators. Statistics on the number of alerts per subject taking 5 or more medications were compiled via individual searches on each subject’s drug regimen.

### Drug-drug interactions involving Cytochrome P450 metabolizing enzymes

The classification of drugs as CYP450 inhibitors, inducers and/or substrates was assembled from the FDA drug interaction labelling guidance [19] and the Flockhart compilation [20]. We identified all elderly subjects taking an inducer and/or inhibitor plus a substrate for the following CYPs: 1A2, 2C9, 2C19, 2D6, 3A4. No drugs identified in our study of elderly subjects from the 2009–2010 survey were listed as 2B6 or 2C8 substrates in the Flockhart compilation, therefore these CYPs were not included in the analysis. Because the FDA compilation included information on inhibitor or inducer strength, we used it preferentially in our analysis. For inhibitors/inducers reported only in the Flockhart compilation, we assumed their strength was low in our analysis. For NHANES combination medicines (e.g. ezetimibe/simvastatin pill), we identified drug-drug interactions involving either active ingredient in the medicine and treated them in the same manner as interactions involving non-combination drugs (e.g. ezetimibe/simvastatin taken with amlodipine was treated the same way as simvastatin taken with amlodipine). Finally, since some Flockhart entries pertain to drug categories (e.g. glucocorticoids, barbiturates), we mapped them to all drugs in the class as defined in NHAHES.

### Quantifying co-occurrence of prescription use or literature reports for drug pairs

To quantify the extent to which drugs were prescribed together (or discussed in the literature) to a greater extent than expected based on their individual rates, we compared the observed and expected rates for drugs A and B:

Observed co-association / Expected co-association = P(A, B) /P(A)*P(B)

where P(A, B) is the observed probability of A and B occurring together, and P(A), P(B) are the individual probabilities for A and B. If the two drugs are used independently, the ratio equals 1. It exceeds 1 when they are co-prescribed more than expected based on their individual rates, and is less than 1 for the converse. Throughout this manuscript, we refer to this quantity as “enrichment”. This term is also denoted as “lift” or “interest” in association rule mining and has been used to identify the rate at which diseases co-occur more than expected from an epidemiology survey [21].

### Network generation

Networks of enriched co-prescription rates (i.e. co-prescription that exceeds the expected frequency for independent drug use) were created using Cytoscape 3.1.0, using the native force directed layout algorithm (unweighted). Node positions were adjusted manually to improve clarity. To increase our ability to detect significant co-used drug sub-classes, we aggregated drugs using the NHANES subclasses RXDICN1B and RXDICN1C (e.g. “diuretics” and “thiazide diuretics”). We calculated percentage usage in the US population by pooling individuals taking any drug in the subclass. We compared all pairs of subclasses at both levels (i.e. all combinations of RXDICN1B and RXDICN1C terms), except those that were trivially related via the class ontology (e.g. “diuretics” vs. “thiazide diuretics”).

### Text mining of literature

We identified 897 co-prescribed drug pairs in 0.1% or more of the elderly (and requiring 5 actual NHANES subjects surveyed) in either of the 2007–2008 or 2009–2010 surveys. These pairs pertained to 133 individual drugs, which were used for querying Medline abstracts from 2004 to July 2014 using the text mining application I2E [22]. The NCI and MeSH ontologies were used for expansion of drug names to include their synonyms; a match to any part of the Medline record was allowed (title, abstract, MeSH, etc). For single agent drugs, we simply supplied the NHANES drug name and used the I2E functionality for synonym expansion. For combination drugs (e.g. acetaminophen/hydrocodone) we searched for the drug pair (using I2E functionality for allowing different ordering and/or delimiters) and the brand name (e.g. Vicodin). We identified any abstract mentioning a drug (i.e. not restricting to articles pertaining to adverse events), since the objective was to quantify the rate at which drug pairs are co-discussed in the medical literature.

## Results

### Prescription Trends

**Fig 1:**
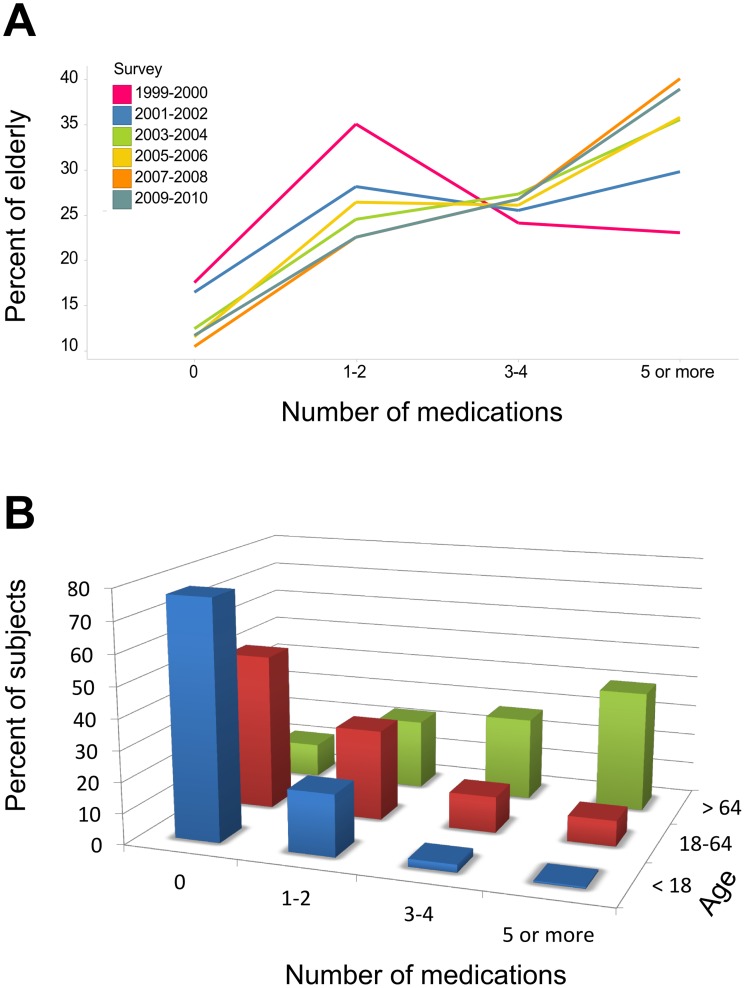
Prescription trends by age and NHANES survey period. A) Percent of elderly subjects taking a given number of medications for each of the past five NHANES data releases. B) Number of prescriptions per person grouped by age for 10,537 subjects from the 2009–2010 NHANES survey. The average number of drugs prescribed per subject are 0.4, 1.4 and 4.1 for subjects <18 years, between 18 and 64 years and ≥65 years old, respectively.

We examined the rate of polypharmacy in various age groups. As expected, the number of prescribed medications per subject increased with both time and age (we use the terms “prescribed” and “taken” interchangeably; NHANES interviewers asked whether medications were being taken, and requested to see containers). Over the past 10 years, the percentage of elderly subjects taking two or fewer medications decreased, while the percentage of subjects taking five or more medications has steadily increased (Fig. 1A). Subjects in the <18 and 18–64 age groups were most frequently prescribed zero medications, and subjects aged 65 and older were most frequently prescribed five or more medications (Fig. 1B). The average number of medications for the elderly population was 4.1 medications/subject, with a maximum of 20 medications in two subjects. Because polypharmacy was most common in the elderly, we focused on this subset for further investigations of polypharmacy and potential drug-drug interactions.

The 20 most commonly reported medications for all adult and the elderly subgroup are listed in Table 1. Seventeen of 20 medications had a higher prescribing frequency in the elderly cohort relative to the overall adult population. Medications used to treat cardiovascular disease as a class were prescribed at a rate approximately three times more often in the elderly, whereas age-dependent enrichment was not observed with other medication classes, most notably drugs in the pain, respiratory and antidepressant classes.

**Table 1:** Prescription frequency of the top 20 prescribed medications for adults and the elderly (≥65 years old) from the 2009–2010 NHANES survey.

drug	class	% adults	% elderly	ratio
simvastatin	metabolic agents	6.8	19.8	2.9
levothyroxine	hormones/hormone modifiers	6.7	16.0	2.4
lisinopril	cardiovascular agents	6.6	16.6	2.5
metoprolol	cardiovascular agents	5.1	17.1	3.4
omeprazole	gastrointestinal agents	4.4	10.2	2.3
metformin	metabolic agents	4.3	9.7	2.3
atorvastatin	metabolic agents	4.0	10.7	2.7
hydrochlorothiazide	cardiovascular agents	3.4	8.7	2.6
albuterol	respiratory agents	3.3	3.3	1.0
amlodipine	cardiovascular agents	3.2	10.1	3.2
atenolol	cardiovascular agents	2.9	9.2	3.2
furosemide	cardiovascular agents	2.7	10.1	3.8
esomeprazole	gastrointestinal agents	2.2	3.9	1.8
ranitidine	gastrointestinal agents	2.2	3.1	1.4
acetaminophen; hydrocodone	central nervous system agents	2.1	2.0	0.9
potassium chloride	nutritional products	1.8	6.0	3.4
sertraline	psychotherapeutic agents	1.8	3.1	1.8
warfarin	coagulation modifiers	1.7	7.8	4.5
citalopram	psychotherapeutic agents	1.7	1.8	1.1
rosuvastatin	metabolic agents	1.7	3.4	2.1

### Co-prescribing Trends

To determine the frequency at which subjects on multiple medications were prescribed the same mix of medicines, we determined the number of unique drug regimens encountered per 100 subjects using the 2009–2010 survey (Table 2). The individual medications prescribed to the elderly were often unique for subjects prescribed 3 medications, nearly always unique for subjects prescribed 4 medications, and were always unique for subjects on 5 or more medications. The same trend holds when examining drug regimens for all adults, except for those taking one medication (which is mostly explained by contraceptive use) and when examining earlier surveys (S1 Fig.).

**Table 2:** Number of unique drug regimens per 100 subjects taking a given number of medications.

	Unique drug regimens per 100 subjects	
Number of medications	adults	elderly
0	1	1
1	26	53
2	84	90
3	98	98
4	99	99
5 or more	100	100

**Fig 2:**
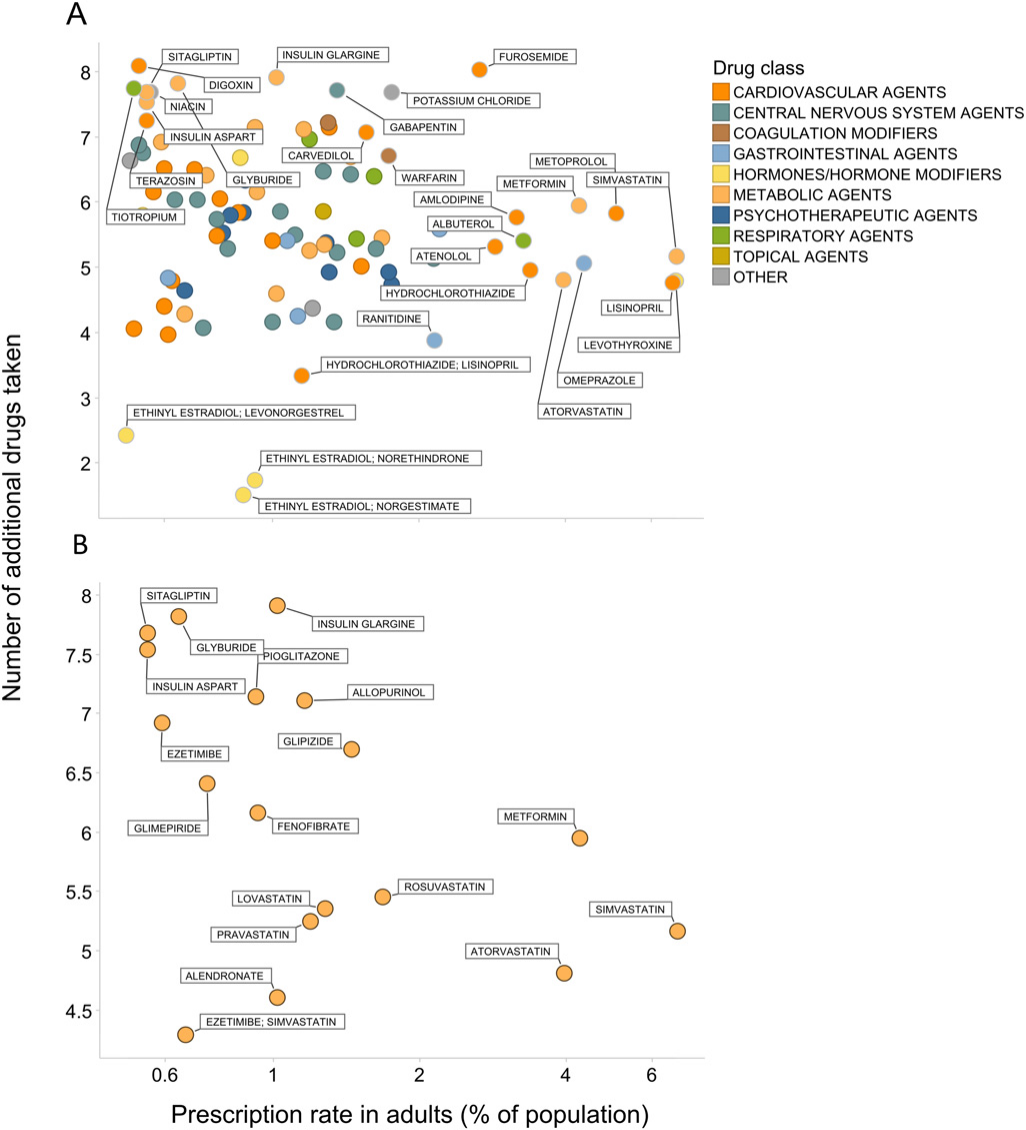
Co-prescription rates relative to drug prescribing frequency. Percent of adult subjects taking a given prescription medication (X-axis) vs. average number of co-taken drugs (Y-axis). Only medications taken by 0.5% of the adult population or more are shown. A) all drugs, B) only metabolic agents. The X-axis is shown on a log scale to improve clarity.

The overall prescription rate for medications differed from corresponding co-prescription rates. For each drug prescribed in 0.5% or more of the adult population, we compared the prescription rate in adults vs. the number of additional drugs taken (Fig. 2A). On average, the most commonly-taken medications (e.g. statins, omeprazole, albuterol) were co-prescribed with approximately 4 other drugs. Gabapentin and furosemide, while prescribed to fewer adults, were co-prescribed at a significantly higher rate (ca. 7 additional drugs). Among metabolic agents, drugs used to treat diabetes early in the course of disease (e.g., metformin as a first line treatment) had higher rates of overall use but lower rates of co-prescription relative to treatments that tend to be prescribed concomitantly later in the disease course (e.g., thiazolidinediones and insulin; Fig. 2B).

**Fig 3:**
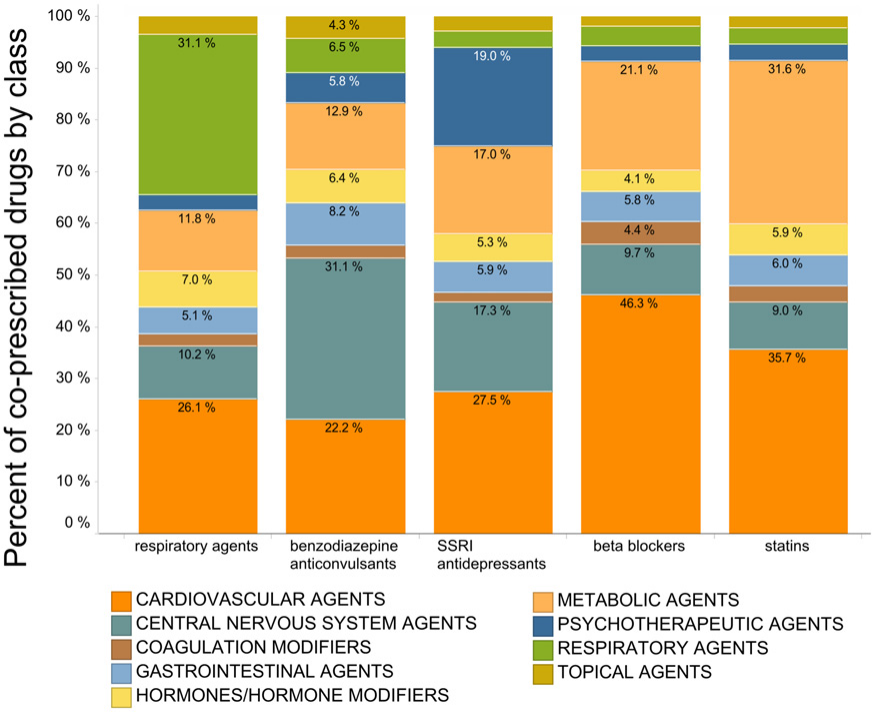
Co-prescription rates by therapeutic indication. Co-prescribed drugs categorized by drug class for all adults in the 2009–2010 NHANES dataset. For the 3 most prescribed drugs in each of the five classes shown, the distribution of classes for additional co-prescribed drugs is shown. Color-coding by therapeutic indication is represented as indicated in legends. For clarity, the drug classes ‘alternative medicines’, ‘anti-infectives’, ‘antineoplastics’, ‘biologicals’, genitourinary tract agents’, ‘immunologic agents’, ‘miscellaneous agents’, ‘nutritional products’, taken by fewer than 10% of elderly subjects, are removed.

We sought to characterize whether co-prescription behaviors were shared across agents within generalized drug classes. For the top 3 medications in each class, we calculated the distribution of drug classes commonly co-prescribed with that medication (S2 Fig.). Within classes, the top 3 prescribed drugs demonstrated similar distributions of co-prescribed medications. However, different classes of drugs showed significantly different distributions of co-prescribed medications, in line with expected co-morbidities (Fig. 3).

**Fig 4:**
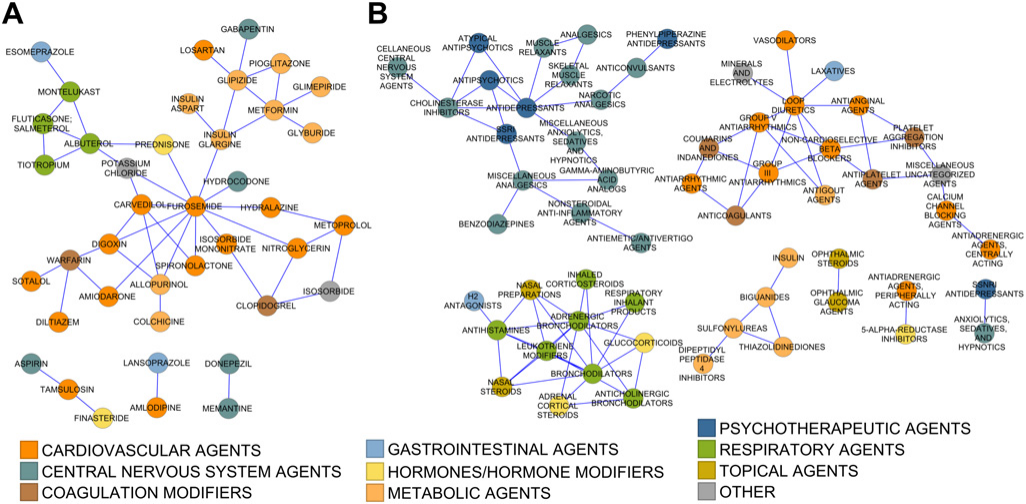
Network representation of coprescription enrichment. Drug pairs (A) or drug subclasses (B) showing enriched co-prescription in the 2007–2008 and 2009–2010 NHANES surveys. Nodes represent drugs or drug subclasses, and edges connect drugs/drug classes with average enrichment ≥3 (i.e 3x more co-prescribed than expected) calculated over two surveys. In addition, the drug/subclass pair must have been observed in 5 or more subjects within each survey (methods).

The discordance between overall and co-prescribing rates prompted us to examine which specific drugs or drug classes were co-prescribed at rates substantially higher or lower than expected based on their individual prescription rates in the elderly. To quantify this, we compared the actual probability of taking two drugs vs. the conditional probability assuming the drugs were used independently. For example, the beta-blocker metoprolol and anti-platelet agent clopidogrel were prescribed together in 2.2% of the elderly, or 3.0 times more than expected if they were used independently (Methods); this is consistent with their use for treating cardiovascular disease. Because many drug pairs were taken by few subjects in the 2009–2010 survey, we calculated enrichment in the elderly for the 2007–2008 and 2009–2010 surveys, and averaged the two values. In addition, we required that at least 5 subjects took the two drugs in each of the surveys to avoid creating spurious associations based on one or two high-weight subjects (the sampling approach used in NHANES gave greater weight to individual non-hispanic whites as representatives of large demographic groups). We created a network visualization showing significant enrichment pairs for both individual drugs and drug subclasses (Fig. 4). The enrichment results for all drug pairs are given in S1 Dataset.

In the network diagram (Fig. 4A), furosemide formed a hub because of its high co-prescription rate. Other drugs with highly enriched co-prescription rates included the anti-diabetic metformin and the mineral supplement potassium chloride. In addition, some less-obvious relationships emerged including 1) furosemide (a loop diuretic) and prednisone (steroid anti-inflammatory) shared a diagram edge despite the fact that they generated an alert on drugs.com for increasing the risk of hypokalemia; 2) glipizide (a sulfonylurea for type II diabetes) and gabapentin prescribed off-label for treating pain from diabetic neuropathy; and 3) amlodipine for hypertension prescribed with the gastric acid suppressant lansoprazole. At the subclass level, expected relationships were observed between co-used drugs for type II diabetes (salmon), respiratory (pink) and cardiovascular disease (green). As expected, a greater number of relationships met the imposed criteria of 5 subjects in each of the two surveys when drugs in a class or subclass were combined. In addition to the comorbidity clusters seen at the drug level (type II diabetes, respiratory and cardiovascular), a significant subclass clustering of CNS and psychotropic drugs emerged.

### Co-discussion of drugs in the medical literature

As an estimate of the availability of clinical knowledge about individual drug-drug interactions, we studied the relationship between co-prescription and co-discussion of drugs in the medical literature. We focused on 133 drugs that were co-prescribed in the elderly at rates of 0.1% or greater in either of the 2007–2008 and 2009–2010 surveys. Medline was searched for each of these drugs using the application I2E [22], which retrieved articles using the NHANES drug name or its synonyms. In total, 186,793 articles were returned, each of which mentioned between 1 and 18 of the 133 drugs (S1 Table). We removed 421 articles discussing more than 5 drugs, as these were predominantly review articles of drug classes (e.g. a review of statins) rather than articles on the co-use of specific drugs. In a manner analogous to co-prescription of drugs, we calculate the proportion of articles describing a particular drug pair, and convert to an enrichment factor which quantifies increased or decreased co-discussion vs. the rate expected if the drugs are co-discussed independently.

**Fig 5:**
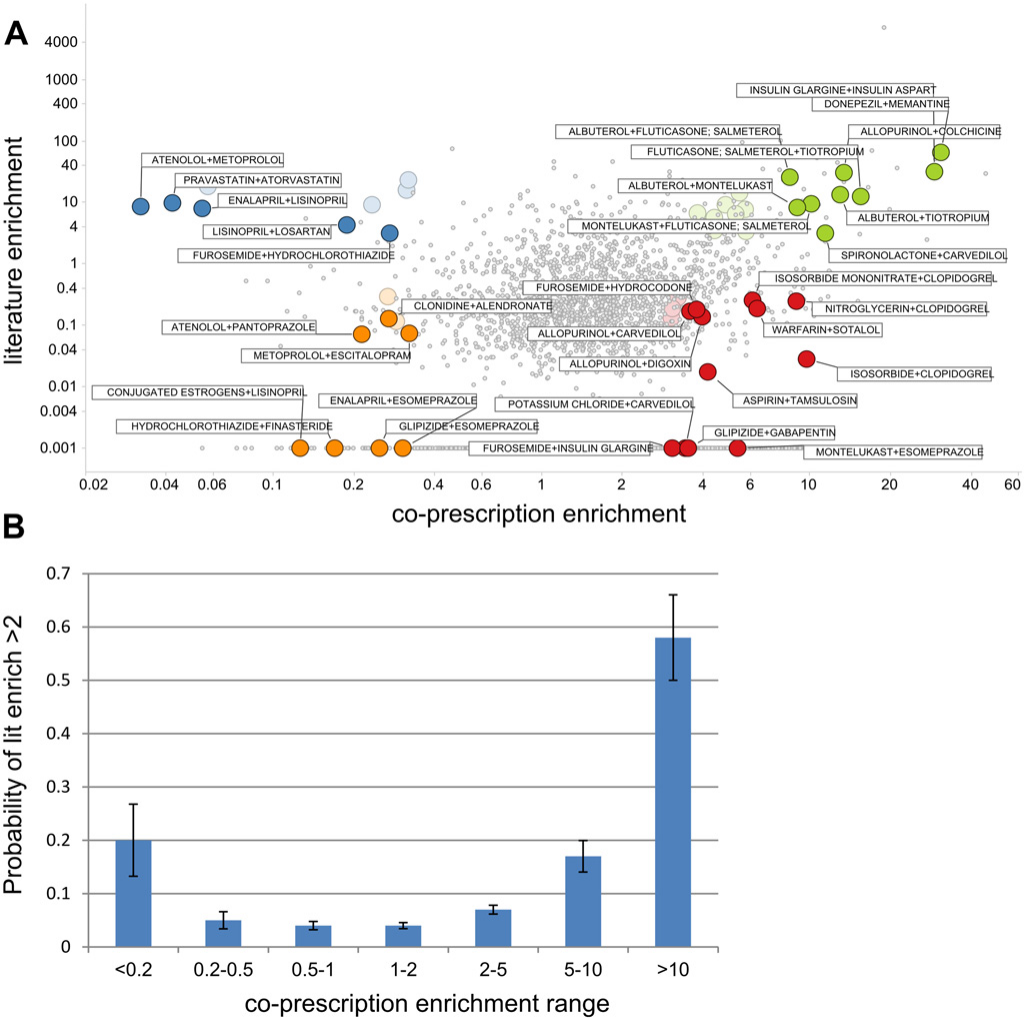
Relationship between enrichment of drug co-discussion in Medline and co-prescription from NHANES 2007–2008 and 2009–2010 surveys. Enrichment quantifies the extent to which a pair of drugs are co-discussed or co-prescribed more (enrichment >1) or less (enrichment <1) than expected if discussed/prescribed independently. A) co-prescription enrichment for 4032 drug pairs, arising from 133 drugs prescribed in ≥0.1% of the population for both surveys (and based on 5 or more actual subjects in each of the last two surveys) vs. co-discussion enrichment in the literature; values shown on log scale and enrichment of 0 in literature (i.e. no Medline records describing both drugs together) is arbitrary shown at 0.001 on the Y-axis. The listed drug pairs exemplify low/high co-prescription and co-discussion. Drug pairs co-prescribed >3x more than expected are exemplified in green and red; in addition to above criteria, these drug pairs are co-prescribed in 0.3 percent or more of the population based on 5 or more actual subjects reporting the use of both drugs in each of the last two surveys. Drug pairs co-prescribed >3x less than expected are exemplified in blue and orange; these drugs have individual prescription rates ≥1% in the last two surveys. B) Probability of co-discussion enrichment >2 when co-prescription enrichment is analyzed in ranges.

We sought to determine whether a relationship existed between the enrichment metric based on co-prescription of drugs from the NHANES surveys and co-discussion in the medical literature (Fig. 5A). We subdivided the drug pairs into 7 ranges of co-prescription enrichment, where enrichment values less than 1 indicate less co-prescription than expected and values greater than 1 indicate more co-prescription. Medical publications were most frequently identified at the two extremes of co-prescription, with medications that had co-prescription enrichment factors of >10 or <0.2 the most likely to be discussed in the literature (Fig. 5B). However, a large percentage of individual drug pairs with co-prescription enrichment ≥ 2 were not mentioned together in any Medline record. Of 106 drug pairs with average co-prescription enrichment ≥ 2 and reported by 5 or more elderly subjects across the 2007–2008 and 2009–2010 surveys, 16 pairs were not mentioned together in any Medline record and a further 38 demonstrated co-discussion enrichment values less than 0.5 (S1 Dataset). In contrast, drug pairs co-prescribed less than expected can generate enriched co-discussion due to known safety issues. Under these circumstances, it would be expected that the discovery and mechanistic study of drug safety increases the rate of publication for problematic drug pairs, which should lead to increased awareness and, therefore fewer prescriptions being written, for that pair. For example, increased risk of hyperkalemia results from combining spironolactone, a potassium-sparing diuretic, and lisinopril, an angiotensin converting enzyme inhibitor. These drugs have literature enrichment of 3.0 and are co-taken by 11 elderly subjects in the two surveys utilized in this analysis. Increased co-discussion can also occur because the literature discusses them as alternative treatments in a class with no expectation of combining them: omeprazole and esomeprazole, enantiomeric proton-pump inhibitors, have literature enrichment of 16 and were co-taken by 3 elderly subjects in the 2009–2010 survey.

### Potential Drug Interactions

Drug interaction alert systems designed to inform prescribers of proven and predicted pharmacodynamic and pharmacokinetic drug interactions have been in place for many years. We identified 672 drug pairs taken by 0.1% of the elderly population that were reported in 5 or more subjects from the 2009–2010 survey. These drug pairs were cross-referenced with a publicly available drug alert system (Drugs.com) to determine the frequency and type of drug interactions expected in real practice. Multiple drug alerts were triggered for subjects in this category (Table 3). Of the 172 subjects on five medications, the average number of ‘alert’ interactions was 3.3 per subject and increased with the number of medications used (S2 Dataset).

**Table 3:** Drug Alerts in the Elderly.

	Number of medications taken					
	5	6	7	8	9	10
Number of subjects (% of elderly)^a^	172 (11.0)	114 (8.0)	100 (5.9)	70 (4.6)	50 (2.9)	39 (2.6)
Total alerts/person	3.3	5.1	6.5	8.9	10.7	13.7
Major alerts/person	0.3	0.5	0.5	0.6	1.2	1.6
Moderate alerts/person	2.4	3.8	5.0	6.9	7.9	9.9
Minor alerts/person	0.6	0.9	1.0	1.4	1.6	2.1

Number and type of drugs.com alerts for 2009–2010 NHANES subjects ≥65 years old taking five or more medications simultaneously.

^a^ the percentage of elderly taking this number of drugs estimated using the survey weights

**Fig 6:**
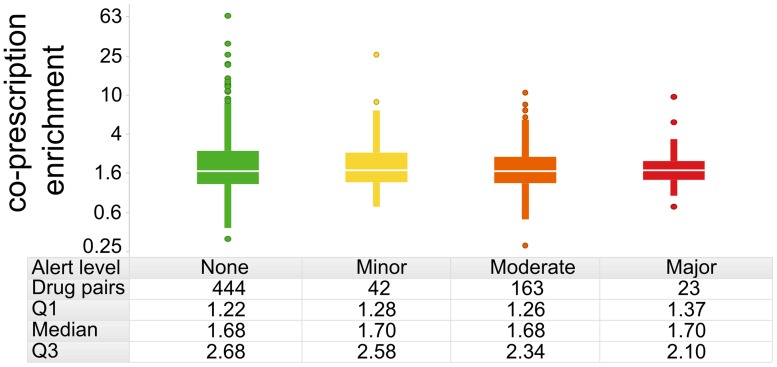
Enrichment in co-prescription rate vs. Drugs.com alert level. The enrichment metric calculates whether a given drug pair is prescribed at a different rate than expected if the two drugs are used independently. For drug pairs that are actively avoided, enrichment values should be < 1. The enrichment metric is plotted on a log10 scale to normalize its distribution. None of the pairwise comparisons across alert categories are statistically significant (p > 0.05 via two-sided t-tests).

We returned to the enrichment metric used to examine whether pairs of drugs were co-prescribed to a greater or lesser extent than expected if they were used independently. Enrichment values < 1 indicate drug pairs that were actively avoided. Fig. 6 shows the distribution of enrichment for drug pairs with major, minor, moderate or no alerts. From these data it can be seen that drugs with alerts were neither more or less frequently co-prescribed than drugs generating no alerts. We detected no statistically significant difference in prescribing enrichment relative to alert level (two-sided t-test p-values > 0.05 for all alert level pairs), indicating that overall prescribing did not take into account drug alerts, at least according to the Cerner-Multum alert system, which is used within Drugs.com. Likewise, we observed no significant differences when using alerts from Micromedex, a subscription-only resource (results not shown).

**Fig 7:**
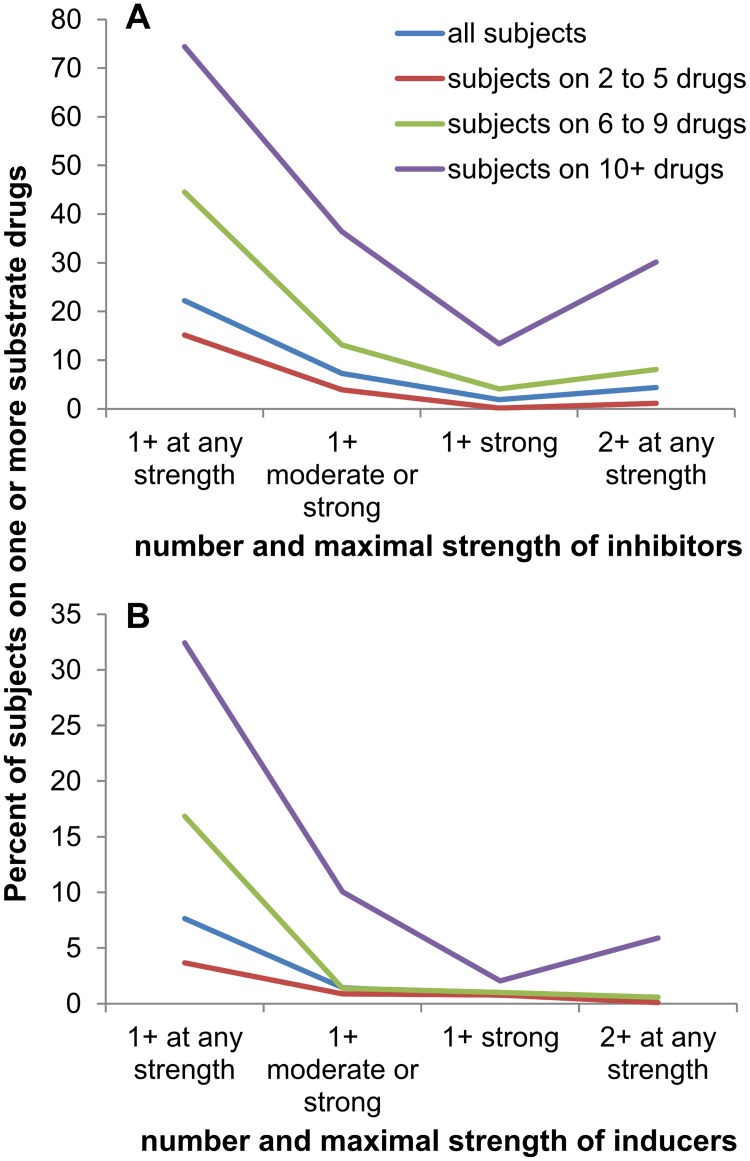
Prevalence of potential drug-drug interactions. Percentage of elderly subjects taking a given number and/or strength of CYP inhibiting or inducing drugs in addition to one or more substrates for the affected CYP; 2+ at any strength indicates that a subject is taking two or more drugs which inhibit or induce CYPs metabolizing a given substrate. A) inhibitors, B) inducers

Alert systems, such as those available on drugs.com, provide pharmacokinetic information on potential drug-drug interactions (DDI) arising from combining cytochrome P450 inhibitors/inducers and substrates. We studied the frequency of potential CYP-mediated DDIs using classification of drugs from the FDA website and the Flockhart compilation (methods). Specifically, we examined the percentage of elderly subjects taking one or more CYP inhibitors or inducers simultaneously with one or more substrates of the given CYP (Fig. 7). As expected, increased polypharmacy increased the probability of potential CYP-mediated DDIs. Less expected perhaps, the percentage of elderly taking multiple inhibitors of a given CYP was 4%, which rose to 30% among those taking 10 or more medicines. Put differently, 0.11 drugs per elderly subject were potentially affected by two or more CYP inhibitors, rising to 0.38 among those on 10 or more medicines. This phenomenon is exemplified by subject #55046, who was taking the moderate CYP2C19 inhibitors fluoxetine and omeprazole, while also taking its substrates warfarin and citalopram.

## Discussion and Conclusion

A simple analysis of prescribing rates using the NHANES dataset demonstrates that a significant percentage of the US population are prescribed multiple medications simultaneously, and that this number has increased over time and with subject age. The types of medications most frequently prescribed are consistent with prescription rate analyses published previously, and reasons for increased polypharmacy include the expansion of insurance coverage, rising prevalence of chronic disease, and population aging [9]. The prescribing rates of certain medication classes increase disproportionally with age, with medications used to treat cardiovascular disease being prescribed at a rate approximately three times higher in the elderly. Metabolic disease-preventing medications are increasingly prescribed in the elderly as well, with one in five elderly patients prescribed simvastatin and nearly 40% of elderly patients prescribed a drug in the statin class. Other medication classes, most notably analgesics and antidepressant medications, are used at similar rates across adult age groups, demonstrating that the progress of patients into polypharmacy is becoming increasingly common. This work expands on analyses of individual drug prescription rates by examining co-prescription trends as a means to better understand the complexities of polypharmacy and the predicted frequency and impact of multi-drug interactions. Our co-prescription rate analyses shows that drug combinations are not simply the sum of prescribing rates for individual medications, and when combined with drug interaction data in a representative sample of the US population, can inform future prescribing, especially in lieu of recent efforts toward reducing polypharmacy as a best practice in treatment [23].

The enrichment metric used in this work quantifies the extent to which drugs are co-prescribed more or less than expected given their individual prescription rates. Commonly prescribed drugs that exhibit highly enriched co-prescription rates include the anti-diabetic metformin and the mineral supplement potassium chloride. Enrichment analysis also identifies drugs that are commonly co-administered for treating a single disease, such as albuterol plus fluticasone/salmeterol and tiotropium. In addition, drugs that are prescribed as secondary (add on) therapy are co-prescribed more frequently than expected. This is exemplified by furosemide, a hub drug in the co-prescription enrichment network. The observation that approximately 50% of drug pairs co-prescribed more than expected are co-discussed in the literature substantially less than expected underlines the need for further study. Some of these associations may arise from co-morbidities that are not fully appreciated. When drugs are co-discussed in the literature, the subject may not pertain to drug-drug interactions or safety issues (different treatment options, co-morbidities, etc.). On the other hand, the absence (absolute or relative) of articles describing drug pairs is not equivocal—these represent genuinely under-studied drug pairs.

There are clear and interesting trends when studying co-prescription rates of highly prescribed drugs such as furosemide. However, investigating co-prescription rates in less frequently prescribed medications can also uncover important trends. Gabapentin, cyclobenzaprine, lorazepam and diazepam are prescribed more frequently as a percentage of overall co-medication with other CNS acting drugs. These data are significant in light of the disproportionate number of predicted of drug-interactions often observed within a given class of drugs. In this instance the predicted interactions are often due to CNS drug inhibition or metabolism by the enzyme CYP2D6 as elaborated upon below.

Co-prescribing rates follow patterns that trend with comorbidity. Co-prescribed drug distributions are similar within a medication class (e.g. beta blockers; S2 Fig.), but different between classes (e.g. anticonvulsants vs. statins; Fig. 3). However, it can be misleading to view co-prescribing by medication class alone, because each medication class is composed of drugs that modulate multiple targets, and for each drug target multiple drug choices are typically available. For example, there are many choices of antidepressants, the most popular of which are selective serotonin reuptake inhibitors (SSRIs). Prescribers can choose sertraline, fluoxetine, citalopram, paroxetine, or a handful of less frequently prescribed SSRIs to treat depression. Even though fluoxetine and citalopram are of the same medication class and each produces pharmacodynamic effects via the inhibition of serotonin reuptake, they are markedly different with regard to predicted pharmacokinetic drug-drug interactions. Fluoxetine is metabolized by and a strong inhibitor of CYP2D6, while citalopram is metabolized primarily by CYP2C19. Therefore, when taking into account co-prescribed medications, the within-class SSRI antidepressant choice becomes important. Drug-drug interactions may result when a co-administered CYP2D6 substrate such as tamoxifen is prescribed alongside fluoxetine, or a CYP2C19 inhibitor such as omeprazole with citalopram. Prescribers frequently substitute one SSRI for another if the first is ineffective or poorly tolerated, and non-pharmacokinetic/pharmacologic factors often drive prescribing habits. If the use of potentially interacting drugs cannot be avoided, adverse clinical consequences may be minimized with individualized dose adjustments guided by clinical response or monitoring plasma drug concentrations, but that approach is used minimally in practice [24].

It has been shown that patients initiating antidepressant treatment receive anxiolytic co-prescriptions at a rate that exceeds the rate of general use [25]. Co-prescription of psychotropic and other CNS-acting medications creates an especially high risk for drug-drug interactions in that these agents are often transported and metabolized by the same polymorphic enzymes. When antidepressant drug levels were measured in the blood of patients in a general practice setting, Kootstra et al. found that 56% of patients were outside of the therapeutic range [26]. This suggests that variability in exposure may be driving the high degree of outcome variability with this class of medications. Drug interactions are predicted to be a very large contributor to this variability, and as can be seen with the data herein, the nature, extent and impact of prescribing trends cannot be overlooked.

Even though co-prescription, when viewed by class, shows similarities in prescribing habits that trend with co-morbidity, the multiple choices of medication within each class can lead to unique medication profiles in individual patients. When analyzing the mix of medications prescribed to the elderly NHANES subjects, we found the individual medications profiles were unique for every subject taking five or more medications. Preskorn et al. also found a high percentage of unique drug combinations when investigating prescribing patterns in Veterans Affairs patients [27]. They attributed this observation to belief that drugs within a specific class were interchangeable. Nearly 40% of patients receiving either fluoxetine or paroxetine were also receiving a drug principally dependent on CYP2D6 for its clearance or that of a clinically relevant metabolite. Findings like these suggest that in many cases prescribers are either unaware of such within-class distinctions or that drug interactions are a secondary concern in decision making.

To explore the frequency of potential drug-drug interactions, we applied publicly available ‘drug alert’ data used by prescribers and pharmacists to co-prescribing data for elderly subjects from the 2009–2010 NHANES survey. One might conclude from the data in Table 3 that most elderly patients with significant polypharmacy would experience frequent adverse events, as the number of moderate and major drug alerts, which include both pharmacokinetic and pharmacodynamics interactions, increases steadily with prescription rate. In hospital settings, increased DDIs are associated with hospital length of stay and cost [10, 28]. In clinical practice, not all potential alerts manifest in altered drug exposure or outcome and most are overridden by prescribers [29], but a significant number have been shown to be clinically important. Duke et al. performed a large scale analysis comparing 13,000 predicted DDIs against a clinical dataset of >2 million patients, and found that nearly 28% of the drug combinations were found in this group. However, only 5.3% of those potentially clinically relevant combinations had actually been tested in clinical pharmacokinetic DDI trials. Of those tested, greater than 60% demonstrated significant changes in drug exposure upon co-administration [12]. We found a similar situation with the combination of amlodipine and simvastatin in 4.0% of the elderly (68 subjects), demonstrating that in real world settings clinically significant drug-drug interactions are occurring. Truly contraindicated drug pairs where sufficient data are available to support causality of adverse drug events, such as simvastatin and gemfibrozil, were found in only 0.44% of the elderly. However, although this may seem to be an infrequent event, this would represent nearly 200,000 patients over the age of 65 in the US alone based on 2012 census estimates. New practices are now available that target improved utilization of drug interaction data [4, 26].

Real world drug interactions are undoubtedly more complex than the pairwise interactions underlying the current prescriber alert systems. For example, we are aware of no studies in the literature testing whether two ‘moderate’ inhibitors of a particular cytochrome P450 that are simultaneously co-prescribed with a substrate of that enzyme produce a greater effect than one potent inhibitor. Multi-drug interaction studies are lacking despite the fact that multiple inhibitors, substrates, or inducers of a single enzyme are often co-prescribed. Our data suggests that this could potentially have a substantial clinical impact in routine practice, as 4% of elderly subjects were prescribed two or more CYP inhibitors simultaneously in addition to a substrate drug. This number of predicted pharmacokinetic interactions is in line with the 6.2% seen by Kerr et al. in elderly Australians [30]. The number of pairwise drug interaction studies necessary to investigate all 690 drugs in the 2009–2010 NHANES data release would be 237,705, and that number would approach infinity when analyses beyond pairwise comparisons are considered. The notion that even the majority of drug interaction combinations could be studied clinically is belied by the rarity of subjects prescribed the same medications in the NHANES survey. These data show that personalized medicine is indeed the norm, as patients taking several medications are experiencing unique pharmacotherapy. Drug tailoring for the polypharmacy patient will require better and more informative approaches to prescribing if it is to be realized in future medicine.

## Supporting Information

S1 Fig Uniqueness of drug regimens in the elderly vs. time. Number of unique drug regimens per 100 elderly subject compiled over 6 NHANES surveys.
(TIF)

S2 Fig Co-prescription rates by therapeutic indication. Co-prescribed drugs categorized by drug class for all subjects in the 2009–2010 NHANES dataset. For each drug, the distribution of drug classes for additional co-prescribed drugs is shown. For example, of the subjects taking 2 or more medications who were taking metoprolol, 33.9% took one or more additional cardiovascular drugs, 26.2% took one or more metabolic drug and 13.9% took one or more CNS drugs, etc. Color-coding by therapeutic indication is represented as indicated in legends. Co-prescription of most highly prescribed medications for beta blockers, respiratory agents, benzodiazepine anticonvulsants, and SSRI antidepressants For clarity, the drug classes ‘alternative medicines’, ‘anti-infectives’, ‘antineoplastics’, ‘biologicals’, genitourinary tract agents’, ‘immunologic agents’, ‘miscellaneous agents’, ‘nutritional products’, taken by fewer than 10% of elderly subjects, are removed.
(TIF)

S1 Table Number of abstracts containing a given number of drugs. (PDF)

S1 Dataset Unique drug pairs from elderly subjects in 2009–2010 survey. All percentages and subject counts calculated on elderly subset only. To find all pairs involving a given drug, columns B and G must both be searched.
(XLS)

S2 Dataset Subjects analyzed from 2009–2010 survey with results of drugs.com search for each individual subject aged 65 years or more taking between 5 and 10 drugs. (XLS)
