# Supplementary figures and images for: Co-Prescription Trends in a Large Cohort of Subjects Predict Substantial Drug-Drug Interactions
Source: PLoS One. 2015 Mar 4;10(3):e0118991. doi: 10.1371/journal.pone.0118991 (PMC4349653; doi:10.1371/journal.pone.0118991)

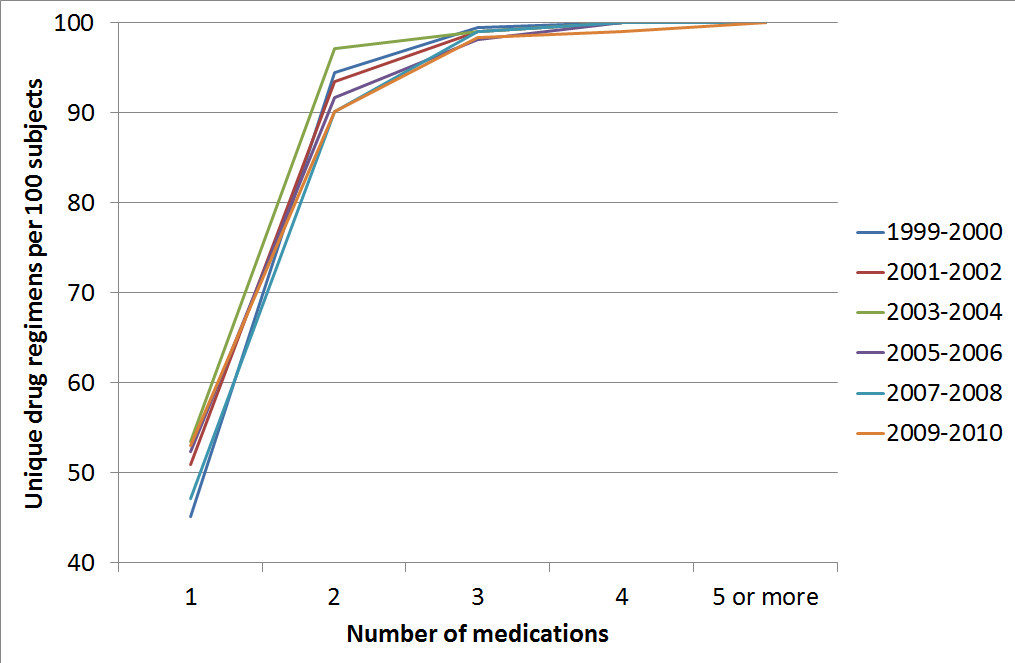

Supplement: S1 Fig — Number of unique drug regimens per 100 elderly subject compiled over 6 NHANES surveys. (TIF) [file pone.0118991.s001.tif]

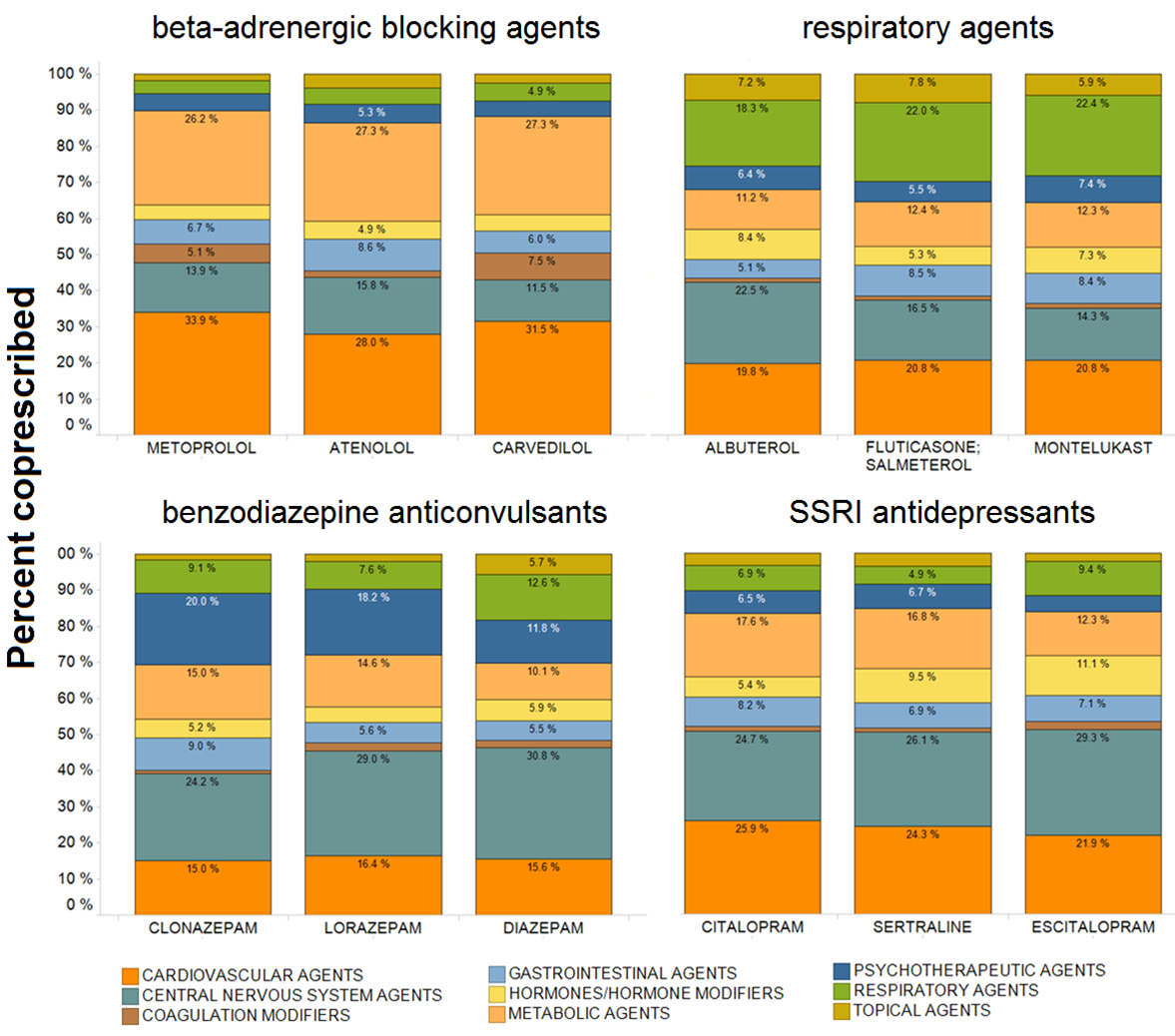

Supplement: S2 Fig — Co-prescribed drugs categorized by drug class for all subjects in the 2009–2010 NHANES dataset. For each drug, the distribution of drug classes for additional co-prescribed drugs is shown. For example, of the subjects taking 2 or more medications who were taking metoprolol, 33.9% took one or more additional cardiovascular drugs, 26.2% took one or more metabolic drug and 13.9% took one or more CNS drugs, etc. Color-coding by therapeutic indication is represented as indicated in legends. Co-prescription of most highly prescribed medications for beta blockers, respiratory agents, benzodiazepine anticonvulsants, and SSRI antidepressants For clarity, the drug classes ‘alternative medicines’, ‘anti-infectives’, ‘antineoplastics’, ‘biologicals’, genitourinary tract agents’, ‘immunologic agents’, ‘miscellaneous agents’, ‘nutritional products’, taken by fewer than 10% of elderly subjects, are removed. (TIF) [file pone.0118991.s002.tif]
