# Supplementary material for: Co-Prescription Trends in a Large Cohort of Subjects Predict Substantial Drug-Drug Interactions
Source: PLoS One. 2015 Mar 4;10(3):e0118991. doi: 10.1371/journal.pone.0118991 (PMC4349653; doi:10.1371/journal.pone.0118991)
Supplement: S1 Table — (PDF) [file pone.0118991.s003.pdf]

Supporting Information Table S1. Number of abstracts containing a given number of drugs

Number of drugs in Medline record[a]	Medline record count
1	151116
2	26206
3	6390
4	1963
5	697
6	195
7	101
8	51
9	28
10	19
>10	27

[a] from the 133 subset of NHANES drugs used for text mining; see methods
